# Extravagance in the Training School

**Published:** 1909-06-05

**Authors:** 


### Nursing Administration.

### EXTRAVAGANCE IN THE TRAINING SCHOOL.

There are grave difficulties in regard to the practice of small economies in institutions. Expenditure on details which in a private house would appear the height of extravagance is necessary in hospitals, because time must be saved, and time is far more costly than lint. In the use of materials the quantity must be sufficient, with a margin, and it obviates difficulties to use the best. And so nurses, after they leave their training school, incur the imputation of being extravagant persons, and few who have known illness in their own houses would be prepared to dispute this generalisation. Is it quite impracticable under the admitted drawbacks of the big institution to teach probationers the meaning of economy? It is one of the anomalies of hospital life that a probationer may be trained in an institution wherein strict economy is practised as a duty by matron and sisters, and may yet gain no insight in her pupil stages into the economic laws which govern the establishment. She may be drilled in avoidance of waste, and this in itself ought to constitute no mean preparation for the practice of economy. Yet she will know nothing about the comparative cost of the articles she uses familiarly, and in private practice she will incline naturally to believe that the most costly preparations must as a matter of course be supplied for her patient's use. Deliberate waste is one thing; lavish use of expensive things in a case of life or death is another. All nurses reprobate the first, at any rate verbally, and no formula is more constantly on the lips of those whose conversation consists of conventional tags than the expression, "If there is one thing I cannot stand, it is waste." Yet with these very words still echoing in the air, the nurse proceeds to use four times more than is necessary of a dressing which has cost four times as much as it need have done, and is unconscious all the time that she is belying her pet axiom.

Would it not be possible to teach the probationers from the beginning the cost of the things they use? In well administered institutions an effort is made to bring all the workers in the ward to a perception of the quantities they use by circulating reports each month of the articles given out under various headings, of the breakages, etc. These returns are passed from one to another, and Sister A has to compare her own rate of consumption with that of Sisters B and C. Nothing can bring home to a careless probationer her iniquities so conclusively as to let her perceive by means of such a form the extent to which isolated acts of negligence have raised the average of the ward. The sense that each deed tells in the general record of the hospital is the sense that generates responsibility. But in these returns a bare enumeration is exclusively relied upon to produce effect. Does anyone in the ward know or care how much these articles cost which were so unfortunately broken last month? Has the probationer been set to compute the money value of her depredations upon the charity? And, again, when some bandage has been (to quote Dr. Jane Walker) "ruthlessly cut," does anyone take the trouble to set the destroyer to calculate the money damage inflicted by her reckless fingers? If probationers are to learn economy they must be taught the cost of every article they use. They must be accustomed to calculate the cost of each patient for every article, until they can tell to a fraction what is involved in doubling unnecessarily the size of a bandage, and have learned "to think in numbers." There is no other way. If they do not learn the value of the things they use while in hospital there is small chance of their gaining a sense of responsibility when attending on private patients, whose friends reluctantly consider it the height of meanness to feel the merest inclination to economise in times of illness, and where goods are ordered in profusion from chemists eager to puff the advantages of patent and costly articles. It may seem that among all the various little things which it behoves the probationer to learn in the course of three years, there is scarcely room for the lessons on prices we have advocated. And yet the number of articles in common use in the wards is not a very long one. When the probationer is padding a splint it would not be a great addition to her labours if she were expected to know its exact cost. When on duty in the out-patient department, it would stimulate her attention to detail if she were told the price of each article used, and were expected to furnish an estimate of the cost of each patient who passed through her hands. Suppose such queries as the following were set at an examination: "State the total cost to the hospital of wasting an average of four inches in length of lint per head on 100,000 patients." "Reckoning that two tablespoonsful of lotion is sufficient for treatment, estimate the quantity in quarts which would be wasted in the course of a year by pouring out double the quantity for use in an average of 30 cases every day. State the cost of this leakage." Would the candidate ever forget the importance of care in little things? We are disposed to think that such care would be greatly stimulated by labelling costly preparations with their price, and by having a table of the prices of things employed in the ward printed and hung up in the ward kitchen. It is far more effective to say "You have thrown away a shilling's worth of milk" than to say "That gallon of milk has gone sour," to warn the probationer "That dressing costs a penny an inch" rather than lavish a flood of vague injunctions against waste. The prevailing custom in all institutions of letting necessaries flow, as it were, from Providence without effort or information is responsible for much of the indifference to economy characteristic of institution-trained individuals. It is a very grave defect in people who have to play their part afterwards in families pressed for means and in the throes of that most costly crisis—a serious illness. Among all the defects which are observable in the system through which private nurses are being prepared in hospitals for their duties to-day, there is none more urgently calling for rectification than this tacit initiation into extravagance

